# Effectiveness and safety of mesenchymal stem/stromal cell for radiation-induced hyposalivation and xerostomia in previous head and neck cancer patients (MESRIX-III): a study protocol for a single-centre, double-blinded, randomised, placebo-controlled, phase II study

**DOI:** 10.1186/s13063-023-07594-5

**Published:** 2023-09-01

**Authors:** Kathrine Kronberg Jakobsen, Amanda-Louise Fenger Carlander, Christian Grønhøj, Tobias Todsen, Jacob Melchiors, Natasja Paaske, Anne Kathrine Østergaard Madsen, Jens Kastrup, Annette Ekblond, Mandana Haack-Sørensen, Mohammad Farhadi, Christian Maare, Jeppe Friborg, Charlotte Duch Lynggard, Christian von Buchwald

**Affiliations:** 1 Department of Otorhinolaryngology, Head and Neck Surgery & Audiology, Rigshospitalet – Copenhagen University Hospital, Copenhagen, Denmark; 2 Cardiology Stem Cell Centre, The Heart Centre, Rigshospitalet, Copenhagen, Denmark; 3 Department of Oncology, University Hospital Zealand, Roskilde, Denmark; 4 Department of Oncology, Herlev Hospital, Herlev, Denmark; 5 Department of Oncology, Rigshospitalet, Copenhagen, Denmark

**Keywords:** Mesenchymal stem cells, Mesenchymal stromal cells, MSC, Xerostomia, Hyposalivation

## Abstract

**Background:**

A predominant side effect of radiotherapy for head and neck cancer is salivary gland hypofunction and xerostomia leading to debilitating oral disorders and impaired quality of life (QoL). Intraglandular mesenchymal stem cell therapy has shown promising results as a treatment for xerostomia.

**Methods:**

This is a randomised, double-blinded, placebo-controlled, parallel-group, prospective, single-centre trial investigating the safety, tolerability, and effectiveness of allogeneic stem cells as a treatment for radiation-induced hyposalivation and xerostomia for previous head and neck cancer patients.

We will include a total of 120 patients who previously have been treated with radiotherapy for a head and neck cancer in Denmark. Participants will be randomly assigned using block randomisation to one of two parallel groups in a 1:1 ratio to receive ultrasound-guided injection of allogeneic adipose-derived mesenchymal stem cell (ASC) (*n* = 60) or placebo (*n* = 60) into the submandibular glands. Placebo will consist of CryoStor10 (BiolifeSolutions), the freeze media for ASCs containing 10% dimethyl sulfoxide (DMSO). The primary endpoint is change in unstimulated whole saliva flow rate. The secondary endpoints are change in stimulated whole saliva flow rate, QoL, and composition of saliva. Further secondary endpoints are safety and immune response (human leukocyte antigen (HLA) response) to the stem cells will be assessed. Patients are evaluated at baseline (before treatment), after 4 months, and after 12 months. All study personnel, except study personnel thawing and preparing the treatment for injection, and participants will be blinded to group assignment. Unblinded study personnel will not participate in the outcome assessment.

**Discussion:**

The trials will investigate the efficacy and safety of ASC injection to the submandibular gland as a potential new treatment for post-radiation xerostomia. We hope the results will pave the way for a clinically relevant treatment to ameliorate patients with xerostomia, a severely hampering condition.

**Trial registration:**

The study is approved by the Danish Data Protection Agency (protocol number P-2020-1164), the National Ethics Committee protocol number: (Protocol number: 1802872), and the Danish Medical Agency (2018-000348-24). The protocol was registered at the ClinicalTrials.gov database (NCT04776538).

**Supplementary Information:**

The online version contains supplementary material available at 10.1186/s13063-023-07594-5.

## Introduction

### Background and rationale

The incidence of head and neck cancer is increasing in the Western World, including Denmark [1–3].

Approximately 80% of patients with head and neck cancer are treated with radiotherapy [4]. Despite the substantial improvement in ionising radiotherapy (IMRT, intensity-modulated radiation therapy), the radiation still leads to significant damage to healthy tissue, including the very radiation-sensitive salivary glands. Particularly, the acinar cells in the salivary glands suffer severe damage from radiation, and as these cells are the principal sites of fluid secretion, one of the most prevalent side effects of irradiation for head and neck cancer is hyposalivation and dry mouth syndrome, xerostomia [5, 6]. Xerostomia can lead to debilitating oral disorders and have a detrimental and lifelong impact on the quality of life (QoL) of the patients [7]. Xerostomia can cause impairment of normal oral functions including talking, chewing, and swallowing, can cause dental caries, and significantly decrease the quality of sleep [5, 8–10]. Currently, only symptomatic treatment is available to patients suffering from xerostomia, and therefore there is an immense, unmet need for new treatment strategies for hyposalivation and xerostomia [11, 12].

With the aim of restoring the salivary gland function in patients suffering from xerostomia due to radiotherapy, we have completed a randomised, placebo-controlled pilot study with 30 patients evaluating the safety and efficacy of ex vivo expanded autologous adipose-derived mesenchymal stem cells (ASCs) for radiation-induced xerostomia [13, 14]. This study showed that the treatment was safe without any serious adverse events. Also, patients treated with autologous ASCs had an increase in saliva production of 33–50%, and compared to the placebo group the patients treated with ASC experienced a significant improvement in their QoL as evidenced by reduced difficulties in eating [14]. However, we observed a substantial individual variation in ASC manufacturing in regard to cell yield, cell quality, and expansion time. We thus recently investigated the safety of allogeneic ASCs for the treatment of xerostomia in a clinical safety and feasible trial [15], including ten patients who received intraglandular injections of allogeneic ASCs. No serious adverse reaction occurred, and the efficacy data was similar to the results from our first trial with autologous ASCs with an increase in saliva production of 46% after 4 months. Patients also gained vital QoL measures regarding dry mouth, sticky saliva, and swallowing [15].

The purpose of this study is to assess the efficacy and safety of treatment with ASCs from healthy donors on radiation-induced salivary gland hypofunction and xerostomia in previous head and neck cancer patients in a randomised, double-blinded, placebo-controlled trial.

### Objectives

The trial aims to evaluate the effectiveness and safety of treatment with ASCs, as compared to a placebo in patients with radiation-induced salivary gland hypofunction.

The primary research hypothesis is as follows: Does treatment with ASCs, as compared to a placebo, lead to improvements in unstimulated whole salivary flow rate in patients with radiation-induced salivary gland hypofunction?

### Trial design

We will conduct a randomised, single-centre, double-blinded, placebo-controlled trial to compare the effectiveness, safety, and tolerability of allogeneic ASCs as a treatment for radiation-induced hyposalivation and xerostomia for previous head and neck cancer patients.

## Methods: participants, interventions, and outcomes

### Study setting

The study will take place at the Department of Otorhinolaryngology, Head and Neck Surgery & Audiology, Rigshospitalet, Denmark. All patients treated for a head and neck cancer in Denmark are eligible for inclusion if they fulfil the rest of the inclusion criteria.

The study protocol follows the SPIRIT guidelines (Standard Protocol Items: Recommendations for Interventional Trials) [16] (Supplementary 1).

### Eligibility criteria

The inclusion criteria are as follows:

1. Are between 18 and 75 years
2. Previously have been treated with radiotherapy +/− chemotherapy for a head and neck cancer
3. Have a 2 years follow-up without recurrence
4. Have a clinically reduced unstimulated whole saliva flow rate between 0.05 and 0.25 ml/min evaluated by sialometry

The exclusion criteria are as follows:

1. Have had any cancer in the previous 4 years (not including the head and neck cancer and basocellular carcinomas)
2. Receive xerogenic medications
3. Have a penicillin or streptomycin allergy
4. Have any other diseases of the salivary glands, e.g. Sjogren’s syndrome or sialolithiasis
5. Previously had submandibular gland surgery
6. Previously had treatment with any type of stem cells in the saliva glands
7. Are pregnant or had a planned pregnancy within the 4 months study period
8. Are breastfeeding
9. Have been smoking within the previous 6 months
10. Have a current alcohol abuse (consumption must not exceed 7 units/week for women and 14 units/week for men (Danish National board health alcohol guidelines [17])
11. Have any other disease/condition judged by the investigator to be grounds for exclusion

The criteria for withdrawal from the study are as follows:

1. Pregnancy
2. Infection of the transplanted site
3. Withdrawal of consent from the participant^1^
4. Cigarette smoking
5. Cancer (recurrence or new primary cancer, excluding basocellular carcinomas)

### Interventions

Patients are randomised in a 1:1 ratio to receive one ultrasound-guided injection in each submandibular gland of ASCs or placebo. The injections are done without any anaesthesia or pain medications. ASCs are provided by the Cardiology Stem Cell Centre (CSCC) at Rigshospitalet. The donated adipose tissue will undergo a strict processing protocol for isolation of ASCs according to good manufacturing practice (GMP) in clean room facilities [18]. The cell products will come from a total of 4–5 healthy donors. Only one donor’s cells will be used in each cell vial. Placebo is consisting of CryoStor10 (BiolifeSolutions), the freeze media for ASCs containing 10% dimethyl sulfoxide (DMSO). The frozen suspension of ASCs or placebo will be thawed just before injection and 0.5 mL of ASC or placebo are injected into each submandibular gland. Patients receiving ASCs will receive a dose of 25 × 10^6^ cells per gland. Injections are carried out using a sterile 1 ml syringe and a sterile 23G (0.6 mm × 60 mm) needle on each side. A trained project nurse will thaw the frozen suspension of ASCs or placebo just before injection, and the syringes are covered in sterile green tape without information about the content of the syringes, to ensure that neither the patients nor the study staff can see the suspension injected. The injection of ASC or placebo is performed ultrasound-guided by free hand by a trained investigator (doctor) using a linear 9–18 MHz transducer. Patients are placed supine and the neck is slightly rotated and extended. The superficial part of the gland will be visualised, as being the part of the gland located between the digastric muscles and superficial to the mylohyoid muscle. The needle is placed in the gland from the anterior part towards the posterior, in a plane parallel to and superficially to the mylohyoid thus avoiding risk to the lingual artery. Injections are performed during a slow retraction of the needle placing the relevant volume in the gland.

### Outcomes

The primary endpoint is to evaluate the impact of ASC injections on unstimulated salivary gland function compared to placebo in patients with previous head and neck cancer. The change is evaluated from baseline to month four.

Secondary endpoints are as follows:

To compare the effect of ASC injection, relative to placebo, from baseline to month four on all of the following outcome measurements:

1. Change in stimulated salivary gland function.
2. Change in the patient-reported outcome of xerostomia: The European Organisation for Research and Treatment of Cancer Quality of Life Questionnaire, Head and Neck-35 (EORTC QLQ-H&N35):
  - Domains for dry mouth (HNDR)
  - Domains for sticky saliva (HNSS)
  - Domains for swallowing (HNSW)
3. Change in the patient-reported outcome of xerostomia: Xerostomia Questionnaire (XQ).
4. Change in the composition of saliva
5. Safety evaluated by the development of treatment-related adverse events, serious adverse reactions, or death
6. Immune response to allogeneic ASC measured by the development of de novo HLA

Tertiary outcomes are as follows:

The change in unstimulated whole salivary flow rate, stimulated whole salivary flow rate, patient-reported outcome of xerostomia evaluated by two questionnaires (EORTC QLQ-H&N35 and XQ), composition of saliva, safety, and immune response in patients receiving ASC injection compared to placebo is further evaluated at 12 months.

### Participant timeline

Patients who are interested in participating in the project will initially be contacted by phone by the investigators for inquiries about their interest in participating in the research project, and to clarify any initial questions. If patients are interested in participating in the project, they are invited to a screening visit at the Department of Otorhinolaryngology, Head and Neck Surgery, and Audiology, Rigshospitalet. Before the visit patients will receive written information regarding the study, the pamphlet “Forsøgspersoners rettigheder i et sundhedsvidenskabeligt forskningsprojekt” (“Rights of test subjects in a health scientific research project”) [19], and the pamphlet “Før du beslutter dig” (“Before deciding”) [20]. Patients are further informed that they have the right to bring a counsel to the screening visit.

The screening visit is conducted by the principal investigator or a trained sub-investigator (doctor). At the screening visit, patients will initially fill out a consent form. When the consent form is completed, inclusion- and exclusion criteria are reviewed with the patients. Patients will have their medical history evaluated, and undergo an ear, nose, and throat examination including an ultrasound exam of the submandibular salivary glands and the neck. If patients meet inclusion criteria so far, they will have whole saliva flow measured by sialometry (Supplementary 2), have blood samples collected and analysed for relevant kidney, infection, and liver parameters, s-HCG when relevant, and for the presence of HLA antibodies. Furthermore, patients will fill out QoL questionnaires (Supplementary 3). Saliva samples collected during the sialometry will be saved for chemical analyses and analyses of saliva composition (Supplementary 4). If patients are eligible for inclusion, they are randomised in a 1:1 ratio to receive ultrasound-guided injections of either ASCs or placebo in the submandibular glands no later than 90 days after the baseline visit. If patients fulfil one or more exclusion criteria they will not be included in the study hereafter and will not be randomised. Their data including the salivary samples will not be saved.

**Fig. 1:**
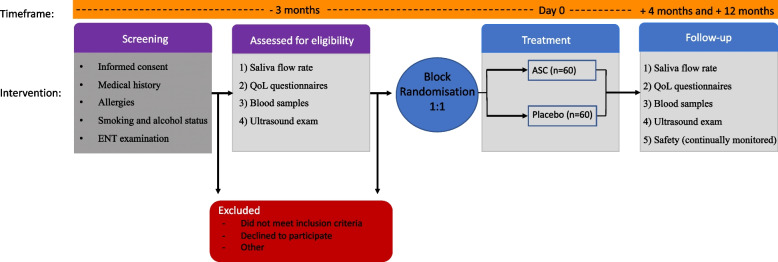
Flowchart of study design. Abbreviation: ENT, ear-nose-throat; QoL, quality of life

**Fig. 2:**
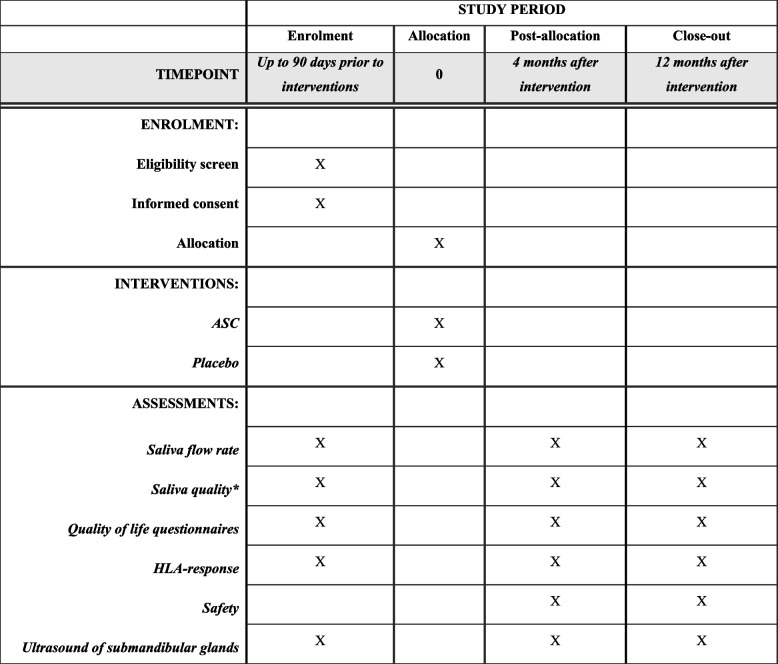
SPIRIT figure

Patients are followed up after 4 month (+/− 14 days) and after 12 months (+/− 4 weeks). At the follow-up visits, patients will undergo saliva flow measurements, have analyses of saliva composition, have blood samples taken (for HLA antibodies), fill out QoL questionnaires, and have an ultrasound exam of the submandibular salivary glands. Lastly, all adverse events are monitored throughout the study period, from the time of treatment until the last day of follow-up (Fig. 1). When all study participants have completed the 4-month follow-up data will be unblinded. All data will be documented on-site in a secure web database, REDCap (Fig. 2).

### Sample size

From our previous study MESRIX-I we assess to increase saliva production for whole unstimulated whole saliva flow rate (in ml/min) by about 33% or in absolute numbers from 0.125 to 0.155 after 4 months. The power calculation is based on a power of 0.8 and an alpha of 0.05. This means that the total number of patients included would have to be 100 (50 in each group). We expect a dropout rate of about 20% and therefore aim to include 120 participants in total.

### Recruitment

Patients are accessible for inclusion if they have been treated with radiotherapy for a head and neck cancer in Denmark. Patients will be recruited from the Department of Otorhinolaryngology, Head and Neck Surgery & Audiology, Rigshospitalet, by referral from other departments, e.g. Oncology Departments in Denmark, or can be self-referred, e.g. triggered by media awareness. 

## Methods: assignment of interventions

### Allocation

A predefined randomisation code is established for all 120 patients according to patient treatment order (1–120) from the start of the trial. The randomisation is done randomly in a 1:1 ratio. The allocation sequence is generated using www.randomization.com. Randomisation is performed in blocks of six, three patients receiving ASCs and three patients receiving placebo to avoid clustering of treatments. Within blocks of six, a balanced use of donors will be applied. The randomisation is done by one person, the allocation manager, at the Cardiology Stem Cell Centre, CSCC, The Heart Centre, Rigshospitalet, and the staff involved in the trial is independent of the randomisation. The allocation manager will store the randomisation table, and the table with randomisation numbers will only be available to one specified person at the CSCC, The Heart Centre, Rigshospitalet, until the end of the trial. This person will not be involved in analysing data related to the study endpoints.

For the purpose of safety and potential unblinding, two sets of sealed envelopes containing the randomisation code for each patient are made. One envelope is locked up at the clinical site at the sponsor and is thereby available for the investigator, and one envelope is locked up at the CSCC production facility. The allocation envelopes will be monitored by the GCP unit during the trials to ensure that they are not opened before the end of the study.

### Blinding

This is a double-blinded study. Both the sponsor, investigators, and study staff (except for staff involved in stem cell preparation and staff involved in bioanalytical analyses) and patients will be blinded to treatment assignment. Data analysts will further use a dataset where the groups are masked. The injection syringes of either ASC or placebo will be identical to ensure blinding.

Emergency unblinding can be performed at any time if it is considered necessary by the principal investigator or the sponsor. Participants are issued with “In case of emergency” cards to be carried at all times during the study including an emergency phone number. If the trial or a single subject is prematurely unblinded the principal investigator will document the reason for unblinding and notify the Danish Medical Agency and the National Ethics Committee.

## Methods: data collection, management, and analysis

### Data collection methods

Data is collected at baseline and at follow-up visits at 4 months (+/− 14 days) and after 12 months (+/− 4 weeks) by the principal investigator or a sub-investigator (doctor). Data are recorded on case report forms (CRFs). Unstimulated whole salivary flow rate and stimulated whole salivary flow rate will be collected by sialometry, and recorded in the CRF immediately after collection. The saliva will be saved and stored in a −80 °C freezer until use. All saliva samples stored in the freezer will be recorded and documented in a freezer log. Patient-reported outcomes will be assessed using the two questionnaires, the EORTC QLQ-H&N35, with a focus on domains for dry mouth (HNDR), domains for sticky saliva (HNSS), and domains for swallowing (HNSW), and the XQ. The patients can answer the questionnaires on-site or at home via unique links sent directly to the database. All adverse events are monitored during the trial. Blood samples will be collected at all the visits to monitor the development of HLA antibodies. The results obtained from the blood samples are directly entered into the patient’s medical record by the laboratory.

Any data insertions or corrections in the CRF will automatically be noted in a data log audit trail. The log audit identifies and records all activities carried out by each individual user, including the data accessed or modified, as well as the patients’ record IDs. A data management plan for the will be completed.

### Data management

All data will be documented on-site in a secure web database, REDCap. Data accuracy will be checked by the principal investigator and the data monitor. The REDCap system will raise queries if discrepancies or errors are spotted.

### Statistical methods

The analysis will be performed on the intention-to-treat population (ITT) and a statistical analysis plan will be completed before the end of the study. The primary endpoint is change in unstimulated whole saliva flow rate. The change in saliva flow rate from baseline to 4-month follow-up visit will be presented as change in mL/min and percentage change. The secondary endpoint of stimulated whole salivary flow rate will also be presented as change in mL/min and percentage change. Patient-reported outcome measurements will be evaluated as the changes from baseline to the 4-month follow-up visit and will be presented as a summary score ranging from 0 to 100. An ANCOVA model will be used to analyse continuous outcome measures (change from baseline). The baseline value will be included as a continuous covariate and the randomised treatment group will be included as a categorical variable. Data will be analysed after all patients have completed their 4-month follow-up time, and after the 12-month follow-up time.

Upon completion of the 4-month follow-up visit for all patients, the data will be unblinded, and an analysis of the 4-month follow-up data will be conducted. Subsequently, the long-term follow-up analysis will take place once all patients have completed their 12-month follow-up.

## Methods: monitoring

### Data monitoring

The trial will be conducted according to the Good Clinical Practice (GCP) guidelines and will be monitored by the GCP unit at the University of Copenhagen.

### Harms

The included patients will be instructed to contact the principal investigator or a trained sub-investigator (doctor) in case they experience any events with possible relation to the trial treatment within the study period. Additionally, at the follow-up visits, all new medical events and any new medications that the patients have received throughout the trial will be thoroughly examined by the principal investigator or a trained sub-investigator. Any adverse events during or after the treatment in the trial will be noted and categorised into severity according to the Common Terminology Criteria for Adverse Events version 5.0 (CTCAE vs 5.0). The onset date, duration, and action taken for the adverse event will be documented.

Adverse events are defined as any untoward medical occurrence in the clinical trial period. All adverse events of CTCAE grade 3 and 4, and incidences considered related to this trial will be reported to the sponsor by the investigator immediately after they are discovered.

A serious adverse event is defined as a medical event that results in the following:

1. Death
2. A serious deterioration in health including:
  - Resulted in life-threatening illness or injury
  - Required hospitalisation or prolongation of existing hospitalisation
  - Resulted in permanent impairment of body structure or body function
  - Resulted in medical or surgical treatment to prevent the above
3. Anything the principal investigator deems to be of clinically serious significance

Any serious adverse events will be reported to the Danish Medical Agency and the National Health Committee along with a report on the patients’ safety once a year. In case of a serious adverse event deemed related to the treatment, the Danish Medical Agency and the National Health Committee will be noted within 15 days or 7 days in case of death or life-threatening events.

Adverse events will be monitored for the first 4 months after the treatment. Serious adverse events will be monitored for the 12-month study period.

### Ethical consideration and risk assessment

Participants will receive either ASCs or a placebo into the submandibular salivary glands by a needle, ultrasound-guided. The risk of infection and bleeding is estimated to be below 0.5%. In our previous studies, none of the 40 participants developed adverse reactions [14, 15]. During the injection of the transplant product (ASCs or placebo), the MESRIX-III participants may experience brief pain. There is a controversial debate regarding the effect of ASCs on cancer growth, as they have been shown to have both inhibitory and stimulatory effects [21–25]. However, multiple clinical trials have been conducted on different participant populations, including local injection of stem cells, involving more than 1000 participants, and no increase in the incidence of cancer has been detected. Extensive research has been carried out on the use of ex vivo expanded stem cells, and no data has been found indicating the malignant transformation of expanded human ASCs [26–28]. A study conducted by Katz et al. on a murine model showed that the injection of a high dose of MSC (240 × 106 MSC/kg) did not reveal any signs of cancer, organ toxicity, or change in body weight after 12 months [29].

### Auditing

The investigators of the trial are responsible for the auditing of the trial and meet every week to discuss the execution of the trial.

## Ethics and dissemination

### Research ethics approval

The study is approved by the Danish Data Protection Agency (protocol number P-2020-1164), the National Ethics Committee protocol number: (Protocol number: 1802872), and the Danish Medical Agency (2018-000348-24). The protocol was registered at the ClinicalTrials.gov database (NCT04776538).

### Protocol amendments

All changes to the protocol will firstly be discussed with the investigators of the trial and will secondly be communicated to the sponsor and other relevant parties. In case of a protocol amendment, this will be submitted to the National Ethical Committee and to the Danish Medical Agency. When the study is completed, the sponsor will inform the Danish Medical Agency within 90 days. Furthermore, all participants will be informed on the completion of the trial.

### Consent or assent

All participants will provide written informed consent before entering the study at the Department of Otorhinolaryngology, Head and Neck Surgery & Audiology. The consent form is collected by the principal investigator or a sub-investigator (a doctor). Patients will receive a copy of the consent form and a patient information sheet.

### Confidentiality

Data collected during the trial will be kept strictly confidential. All data will be documented on-site in a secure web database, REDCap where only the trial team has access. All participants will be allocated to an individual identification number. There are no intentions to share anonymous data with researchers outside the trial unit.

### Declarations of interests

Grants were received from the Candys Foundation (Grant number: 2020-352), and Rigshospitalet (no grant number). The funders will not influence the study design, data collection, data analysis, writing of the report, and the decision to submit the results for publication.

### Access to data

During the trial, the principal investigator and a trained sub-investigator (a doctor) will have access to the full dataset. Only the trial team will have access to the final trial dataset. Furthermore, the monitor will have access to the full dataset during the trial. The patients will provide written consent to participate in the GCP-monitored trial.

### Dissemination policy

The researchers will communicate trial results to participants when the trial is finished. Data will be published in peer-reviewed journals. The dissemination of the data will be coordinated by the principal investigator.

## Discussion

This study will be the largest trial aiming to investigate the efficacy and safety of allogenic ASCs as a treatment for radiation-induced xerostomia. This research project will provide new and original evidence and may help in the treatment of a debilitating oral disorder.

## Trial status

Recruitment of participants began in February 2021 and was finished by February 2023. The last patient/last visit is anticipated to be in February 2024. The trial is currently working to protocol version 2.10 dated 25 January 2023. It was not possible to submit the protocol earlier due to adjustments in the method, and the newest protocol version being finalised by 25 January 2023.

### Supplementary Information

**Additional file 1. **SPIRIT Checklist for *Trials.*

**Additional file 2:** **Supplementary 2.** Sialometry. **Supplementary 3.** Patient-Reported Outcome, quality of life questionaries. **Supplementary 4.** Analysis of saliva.

## Data Availability

The full trial protocol is available on request to the corresponding author.

## Footnotes

1 If participants withdraw consent they are excluded from the study and will not be followed up. The patients will be asked if data collected until the withdrawal can be used in the study or if they wish the data to be deleted. Patients can withdraw consent at any time during the study period.
