# Supplementary material for: Effectiveness and safety of mesenchymal stem/stromal cell for radiation-induced hyposalivation and xerostomia in previous head and neck cancer patients (MESRIX-III): a study protocol for a single-centre, double-blinded, randomised, placebo-controlled, phase II study
Source: Trials. 2023 Sep 1;24:567. doi: 10.1186/s13063-023-07594-5 (PMC10474624; doi:10.1186/s13063-023-07594-5)
Supplement: Supplementary file 1 — Additional file 1. SPIRIT Checklist for Trials. [file 13063_2023_7594_MOESM1_ESM.docx]

|  | **STUDY PERIOD** | | | |
| --- | --- | --- | --- | --- |
|  | **Enrolment** | **Allocation** | **Post-allocation** | **Close-out** |
| **TIMEPOINT** | ***Up to 90 days prior to interventions*** | **0** | ***4 months after intervention*** | ***12 months after intervention*** |
| **ENROLMENT:** |  |  |  |  |
| **Eligibility screen** | X |  |  |  |
| **Informed consent** | X |  |  |  |
| **Allocation** |  | X |  |  |
| **INTERVENTIONS:** |  |  |  |  |
| ***ASC*** |  | X |  |  |
| ***Placebo*** |  | X |  |  |
| **ASSESSMENTS:** |  |  |  |  |
| ***Saliva flow rate*** | X |  | X | X |
| ***Saliva quality*** | X |  | X | X |
| ***Quality of life questionnaires*** | X |  | X | X |
| ***HLA-response*** | X |  | X | X |
| ***Safety*** |  |  | X | X |
| ***Ultrasound of submandibular glands*** | X |  | X | X |
