# Supplementary material for: Effectiveness and safety of mesenchymal stem/stromal cell for radiation-induced hyposalivation and xerostomia in previous head and neck cancer patients (MESRIX-III): a study protocol for a single-centre, double-blinded, randomised, placebo-controlled, phase II study
Source: Trials. 2023 Sep 1;24:567. doi: 10.1186/s13063-023-07594-5 (PMC10474624; doi:10.1186/s13063-023-07594-5)
Supplement: Supplementary file 2 — Additional file 2: Supplementary 2. Sialometry. Supplementary 3. Patient-Reported Outcome, quality of life questionaries. Supplementary 4. Analysis of saliva. [file 13063_2023_7594_MOESM2_ESM.docx]

**Supplementary 2. Sialometry**

The most common method for evaluating the salivary gland function is sialometry. Sialometry is a direct measurement of the flow rate in either unstimulated or stimulated state of secretion. This test is valuable since it is easily quantifiable ^1^. Whole saliva is the secretions from the major and minor salivary glands, which are mixed in the oral cavity. A correct determination of this value is crucial for the assessment of treatment outcomes in this project.

For assessment of the saliva flow rate, whole saliva will be collected between 8 a.m. and 5 p.m. (each participant will have *all* the saliva tests done at the same time point of the day). Subjects will be prohibited from eating and performing oral hygiene for 1 hour before the collection and from drinking two hours before collection. After being seated upright in a chair, they relax for 5 minutes and are then instructed to make as few movements as possible, including swallowing, during the collection. Unstimulated whole saliva will be collected using the spitting method in which participants spit their saliva into a collection container over a period of 10 minutes ^2^. After the collection of unstimulated saliva, the subjects are instructed to chew on 1 g of paraffin wax. They will be asked to keep their mouths closed during chewing and to avoid swallowing. Participants are instructed to collect the stimulated saliva for 5 min. The salivary flow rate (ml/min) is determined by the increase in weight of the container divided by the collection time in minutes.

**Supplementary 3. Patient-Reported Outcome, quality of life questionaries.**

For evaluation of the participants´ perception of xerostomia, the participants will answer questionnaires in Danish at baseline, after four, and twelve months. The questionnaires are essential tools for estimating the degree of quality of life (QoL) and xerostomia ^3,1^. Patients will complete two questionnaires, the Xerostomia Questionnaire (XQ) and the Quality of Life Head and Neck-35 questionnaire (EORTC QLQ-H&N-35).

**Supplementary 4. Analysis of saliva**

Our pilot study MESRIX-II indicated that adipose-derived mesenchymal stem cells (ASCs) stimulated a variety of biological processes due to upregulation of specific proteins in stimulated whole saliva at four months after intervention, which could potentially be involved in tissue regeneration ^4^. We will characterize the salivary proteome with mass spectrometry based proteomics.

**References**

1. Eisbruch A, Rhodus N, Rosenthal D, et al. How should we measure and report radiotherapy-induced xerostomia? Semin Radiat Oncol 2003;13(3):226–34.

2. Navazesh M, Christensen CM. A Comparison of Whole Mouth Resting and Stimulated Salivary Measurement Procedures.

3. Jensen K, Jensen AB, Grau C. A cross sectional quality of life study of 116 recurrence free head and neck cancer patients. The first use of EORTC H&N35 in Danish. Acta Oncol (Madr) 2006;45(1):28–37.

4. Lynggaard CD, Jersie-Christensen R, Juhl M, et al. Intraglandular mesenchymal stem cell treatment induces changes in the salivary proteome of irradiated patients. Commun Med [Internet] 2022 [cited 2023 Jan 11];2(1):160. Available from: https://pubmed.ncbi.nlm.nih.gov/36496530/
